# A148 CASE REPORT: ASSOCIATION OF HOUSE DUST MITE ALLERGY AND REMISSION OF EOSINOPHILIC ESOPHAGITIS AFTER ELIMINATION OF SHRIMP

**DOI:** 10.1093/jcag/gwac036.148

**Published:** 2023-03-07

**Authors:** J Venner, U Kovaltchouk, P Krongold, L McKibbin

**Affiliations:** 1 Gastroenterology; 2 Clinical Immunology and Allergy, University of Manitoba, Winnipeg, Canada

## Abstract

**Background:**

Eosinophilic esophagitis (EoE) is a complex, chronic, allergic disease that commonly presents as dysphagia in young adults. Co-occurring allergic or atopic disease, including food allergies and asthma are seen in approximately 70% of cases. Allergy to house dust mite (HDM) tropomyosin, Der p 10, and cross-reactivity to shrimp has been well described. We discuss a presentation of EoE in the setting of positive HDM skin prick testing that subsequently improved with dietary elimination of shrimp, that has never been described in the literature.

**Purpose:**

To highlight an underlying mechanism in EoE and the importance of a multidisciplinary approach to this disease.

**Method:**

A 45-year-old male with a medical history significant for ulcerative colitis (diagnosed two years prior and maintained in deep endoscopic remission with adalimumab) and asthma (with seasonal, rare usage of budesonide and formoterol inhaler) was seen by his outpatient gastroenterologist following three weeks of new-onset dysphagia and subjective intermittent food bolus impaction. The patient was pre-emptively started on daily proton pump inhibitor prior to endoscopy, but discontinued this within less than two weeks as he felt like it induced loose bowel movements. Esophagogastroduodenoscopy (EGD) was performed and revealed proximal esophageal furrows. Histology of esophageal biopsies was compatible with EoE: distal esophagus >100 eosinophils/hpf with eosinophil degranulation, mid and proximal esophagus >25 eosinophils/hpf with eosinophil degranulation. The patient self-identified that his episodes of dysphagia and food bolus impaction were associated with shrimp ingestion, thus he eliminated shrimp from his diet.

**Result(s):**

Two-month clinical follow-up demonstrated symptomatic remission despite no other active treatment or additional food elimination. Consultation with an allergist demonstrated the patient had positive skin prick testing to HDM (*Dermatophagoides pteronyssinus* and *farina*) but negative testing to shrimp, despite clinical improvement with dietary shrimp elimination. Thus his EoE was attributed to HDM cross-reactivity, specifically to the Der p 10 antigen. Follow-up EGD and biopsy to confirm endoscopic and histologic remission with shrimp elimination is scheduled in two months.

**Conclusion(s):**

This is the first case description of a patient with new onset EoE attributed to Der p 10, in the setting of positive skin prick to HDM and negative skin prick to shrimp, with subsequent symptomatic resolution of EoE following dietary shrimp elimination. Shrimp and HDM share a protein, Der p 10. Der p 10 is commonly the culprit antigen in patients that have allergic reactions to HDM and (or) shrimp, but often only demonstrate positive skin prick testing to HDM (with negative testing to shrimp). This case demonstrates a role for involvement of an allergist and skin prick testing in select cases of EoE, as this may result in a relatively simple food elimination and avoidance of further therapeutic interventions.

**Please acknowledge all funding agencies by checking the applicable boxes below:**

None

**Disclosure of Interest:**

None Declared

